# First sero-prevalence of dengue fever specific immunoglobulin G antibodies in Western and North-Western provinces of Zambia: a population based cross sectional study

**DOI:** 10.1186/1743-422X-11-135

**Published:** 2014-07-30

**Authors:** Mazyanga Lucy Mazaba-Liwewe, Seter Siziya, Mwaka Monze, Idah Mweene-Ndumba, Freddie Masaninga, Peter Songolo, Costantine Malama, Elizabeth Chizema, Peter Mwaba, Olusegun A Babaniyi

**Affiliations:** 1 World Health Organization Country Office, Lusaka/Virology Unit, University Teaching Hospital, Lusaka, Zambia; 2 Clinical Sciences Department, School of Medicine, Copperbelt University, Ndola, Zambia/University of Lusaka, Lusaka, Zambia; 3 Virology Unit, University Teaching Hospital, Lusaka, Zambia; 4 World Health Organization Country Office, Lusaka, Zambia; 5 Ministry of Health headquarters, Lusaka, Zambia; 6 Peter Mwaba. Ministry of Home Affairs, Lusaka, Zambia

**Keywords:** Dengue, Seroprevalence, Zambia, North-Western province, Western province

## Abstract

**Background:**

Dengue fever is a tropical infectious disease caused by dengue virus (DENV), a single positive-stranded RNA Flavivirus. There is no published evidence of dengue in Zambia. The objective of the study was to determine the sero-prevalence and correlates for dengue fever specific IgG antibodies in Western and North-Western provinces in Zambia.

**Methods:**

A randomized cluster design was used to sample participants for yellow fever risk assessment. In order to rule out cross reactivity with other flaviviruses including dengue, differential antibody tests were done by ELISA. Data was processed using Epi Data version 3.1 and transferred to SPSS version 16.0 for analysis. Bivariate and multivariate analyses were performed to determine the association of dengue fever with various factors. Unadjusted odds ratios (OR), adjusted odds ratios (AOR) and their 95% confidence intervals (CI) are reported.

**Results:**

A total of 3,624 persons were sampled for dengue virus infection of whom 53.3% were female and 23.9% were in the 5–14 years age group. Most persons in the survey attained at least primary education (47.6%). No significant association was observed between sex and dengue virus infection (p = 1.000). Overall, 4.1% of the participants tested positive for Dengue IgG. In multivariate analysis, the association of age with Dengue infection showed that those below 5 years of age were 63% (AOR = 0.37; 95% CI [0.16, 0.86]) less likely to be infected with Dengue virus compared to those aged 45 years or older. A significant association was observed between grass thatched roofing and Dengue infection (AOR = 2.28; 95% CI [1.15, 4.53]) Respondents who used Insecticide Treated Nets (ITN) were 21% (AOR = 1.21; 95% CI [1.01, 1.44]) more likely to be infected with dengue infection than those who did not use ITNs. Meanwhile, participants who visited Angola were 73% (AOR = 1.73; 95% CI [1.27, 2.35]) more likely to be infected with Dengue virus than those who did not visit Angola.

**Conclusion:**

This study provides the first evidence of dengue infection circulation in both North-Western and Western provinces of Zambia. It is important that surveillance activities for Dengue and diagnostic systems are expanded and strengthened, nationwide in order to capture information related to dengue virus and other flaviviruses.

## Background

Dengue fever is a tropical infectious disease caused by Dengue virus (DENV), a single positive-stranded RNA virus of the family Flaviviridae; genus Flavivirus [1]. Dengue virus infection is classified into dengue with or without warning signs and severe dengue. Dengue with or without warning signs is further classified into probable dengue and laboratory-confirmed dengue. A case of probable dengue would have lived in/travelled to dengue endemic areas and has fever and 2 of the following criteria: nausea/vomiting, rash, aches and pains, tourniquet test positive, leukopenia and any of the following: abdominal pain or tenderness, persistent vomiting, clinical fluid accumulation, mucosal bleed, lethargy/restlessness, liver enlargement of more 2 cm and increase in HCT concurrent with rapid decrease in platelet count. Meanwhile, severe dengue is classified as severe plasma leakage leading to dengue shock syndrome and fluid accumulation with respiratory distress; severe bleeding and severe organ (such as liver, CNS and heart) involvement [1,2]. Without adequate clinical management, mortality stands at 1–5% [3] and usually less than 1% with adequate clinical management [1]. However, severe disease carries a mortality of 26% [3]. The disease is transmitted between people by mosquito species *Aedes aegypti* and *Aedes albopictus*[4]. The main vector associated with Dengue fever is *Aedes aegypti*[5] which is found worldwide between latitudes 35°N and 35°S [6]. Robinson GG. documented presence of *Aedes Aegypti* (L) in Zambia [7].

The disease is now endemic in more than 100 countries in Africa, the Americas, the Eastern Mediterranean, South-east Asia and Western Pacific [8]. It has become common in Africa and amongst travellers from the tropics and subtropics. Dengue fever is common amongst travellers from the tropics and subtropics [9]. Zambia which lies between latitude 8° and 18°S and longitude 22° and 34°E is considered generally of tropical climate [10,11]. However, the contemporary worldwide risk of dengue virus infection is poorly known [9].

The dengue virus in Africa has been traced as far back as 1926 having caused an epidemic in Durban, South Africa [12,13]. Though surveillance for dengue in Africa is poor, it is known that dengue epidemics have increased dramatically since 1980. Most activity has been documented in East Africa and major out breaks in many countries including Seychelles, Kenya, Mozambique, Djibouti, Somalia and Saudi Arabia between the 1970’s and 1990’s [14]. Between 2009 and 2012 outbreaks were reported in more countries in Africa including Cape Verde, Cote d’Ivoire, Gabon, Senegal in West Africa, Djibouti [15,16] and in Kenya and Sudan in East Africa [17]. Most recently in 2013, Africa recorded dengue outbreaks in Angola, Kenya, Seychelles and Tanzania [18].

Zambia had no documented evidence of dengue infection except for a confirmed case of a European traveller/expatriate who was in Zambia between 1987 and 1993 [15]. Travel by air, motor vehicles or foot increases the risk of introducing arthropod-borne virus diseases from endemic to non-endemic areas [19]. There is increasing travel between the dengue endemic neighbouring countries and Zambia [20].

Zambia is a land linked country surrounded by countries endemic with dengue fever including Angola, Democratic Republic of Congo, Tanzania and Mozambique and yet there is no documented evidence of dengue fever. Zambia could be considered a risk area for dengue virus circulation, considering it neighbours dengue endemic countries, has tropical climate and carries the dengue vector *Aedes aegypti*[15,18].

Recently during the Yellow Fever risk assessment conducted in two provinces of Zambia (Western and North-Western provinces), Zambia confirmed presence of dengue fever IgG antibodies in persons participating in the survey. IgG antibodies against dengue are detectable after 10–14 days of onset and once infected; one has life immunity to the specific serotype they were infected with [21]. This paper describes the sero-prevalence and correlates for dengue fever specific IgG antibodies in Western and North-Western provinces in Zambia.

## Methods

### Study site

The study was conducted in Western and North-Western provinces of Zambia. These two provinces were selected by virtue of being classified as low potential risk for yellow fever transmission by a World Health Organisation (WHO) Yellow Fever Technical Working Group in 2010 [22]. Western province borders with Angola and has seven districts divided into 1902 Standard Enumeration Aras (SEAs). T2e population was at 902,974 with a population density of 7.0 [23]. Crop and livestock production as well as fishing were the main economic activities [24]. North Western province borders with Angola on the western side and Democratic Republic of Congo (DRC) on the northern side. The province had 8 districts that were divided into 1178 SEAs. The 2010 census reveals a population of 727,044 with population density of 5.6 [23]. The main economic activity was pineapple growing, with fast growing mining activities in one of the districts, Solwezi [24].

Zambia Demographic Health Survey, 2007 reports 46.9 and 53.2 per cent of the population slept under an Insecticide Treated Net in the previous night to the survey in Western and North-Western Provinces respectively [25].

### Study population

This assessment was carried out among individuals aged nine months or older.

### Sample size, inclusion/exclusion criteria and sampling

The sample size calculation was based on the assumption that the sero-prevalence was 7% based on the study conducted by Robinson [7].

In estimating the sample size for persons aged 5 years or older, the following parameters were considered: a prevalence of 7%, desired precision or confidence interval (d) of +3%, and a design effect (DE) of 2 and an 80% response rate.

Considering sex, we aimed to recruit 700 male and 700 female participants in each province. Assuming an average of 4 persons aged 5 years or older in each household, a total of 12 households in each of the 30 clusters were to be recruited in the survey. The total number of adult persons that would be recruited from each province was 1400.

The sero-prevalence of children below 5 years was about half that for older children, and in estimating the sample size for persons aged below 5 years, the following parameters were considered: a prevalence of 3.5%, desired precision or confidence interval (d) of +3.4%, and a design effect (DE) of 2 and an 80% response rate. A total of 406 children in each province would be recruited for the survey.

Any individual aged 9 months or older and who was a member of a sampled household and resident in the study site for at least seven days was eligible to participate in the survey. Individuals who received YF vaccination in the last ten years to the survey were also eligible to participate in the survey.

Any person, who was either less than 9 months of age, or any person regardless of age, who resided in the study site for a period of less than seven days prior to the survey was excluded from the study. Determining the sero-prevalence in children under the age of nine months raises the risk of false positive results as children under this age may still carry maternal antibodies from immunized or exposed mothers.

The sample was drawn using a two-stage cluster sampling technique using probability proportional to size. A list of the standard enumeration areas (SEAs) in each province constituted the sampling frame. The line lists of the SEAs were provided by the Zambian Government’s Central Statistics Office (CSO). The study was designed to obtain estimates at the provincial level of analysis, and not representing the subdivisions of the province.

### Study variables

#### Dependent variable

A case with evidence of dengue infection exposure was defined as any individual aged 9 months or older whose blood sample was confirmed to have dengue virus exposure through any of the following:

#### Detection of Dengue virus-specific IgG and IgM antibodies

Dengue Fever exposure status was therefore determined by way of laboratory testing as ‘positive’ , for a respondent with laboratory evidence of dengue virus exposure or as ‘negative’ , for a respondent who did not have laboratory evidence of dengue virus exposure.

#### Independent variables

The independent variables included age, sex, occupation, visitation to Angola and/or DRC, use of mosquito preventive measures (ITNs and Insecticide Residual Spray) and type of roofing.

#### Laboratory procedure

About 3 to 5 millilitres of blood was collected by venepuncture into a plain (red top) vacutainer tube and transported on cold chain to the local laboratories for serum separation and storage.

Serum samples were transported on cold chain and thereafter subjected to primary testing (YFV specific IgG and IgM) at the University of Teaching Hospital in Zambia Virology Unit and Institute Pasteur, Dakar (WHO regional reference laboratory (RRL)). All presumptive YFV-specific IgG and IgM samples were subjected to IgG and IgM antibodies testing against other flaviviruses known to cause haemorrhagic fever-like disease including dengue at the RRL. The testing was carried out using IgG capture enzyme-linked immunosorbent assay (ELISA).

### Data management and analysis

Field data were entered in an Epi Data version 3.1 entry screen that had consistency and range checks embedded in it. Further editing was conducted by running frequencies during the analysis stage. Epi data files were exported to SPSS version 16.0 for data analysis. The data was summarized to describe the occurrence of dengue virus exposed individuals in absolute numbers and percentage by place (residence, travel, roof type or work) and person (age, sex, occupation). Bivariate and multivariate analyses were conducted to determine independent factors associated with dengue IgG sero-positivity. Odds ratios were used to estimate the magnitude of associations. Unadjusted odds ratios (OR) and adjusted odds ratios (AOR) and their 95% confidence intervals (CI) are reported. Yates correlated Chi-square and Pearson’s Chi-square were, where appropriate, used to compare proportions at 5% significance level.

### Ethical considerations

Ethical clearance was sought from the Tropical Diseases Research Centre Research Ethics Committee in Ndola, Zambia, and ethical standards were adhered to throughout this study. Informed consent was sought from study participants. Guardians provided assent for the participation of the persons under the consenting age. They were asked to read or have read to them, understand and sign/thumbprint an informed consent form. Responsible adults in the household were identified to give proxy consent on behalf of minors.

## Results

**Figure 1:**
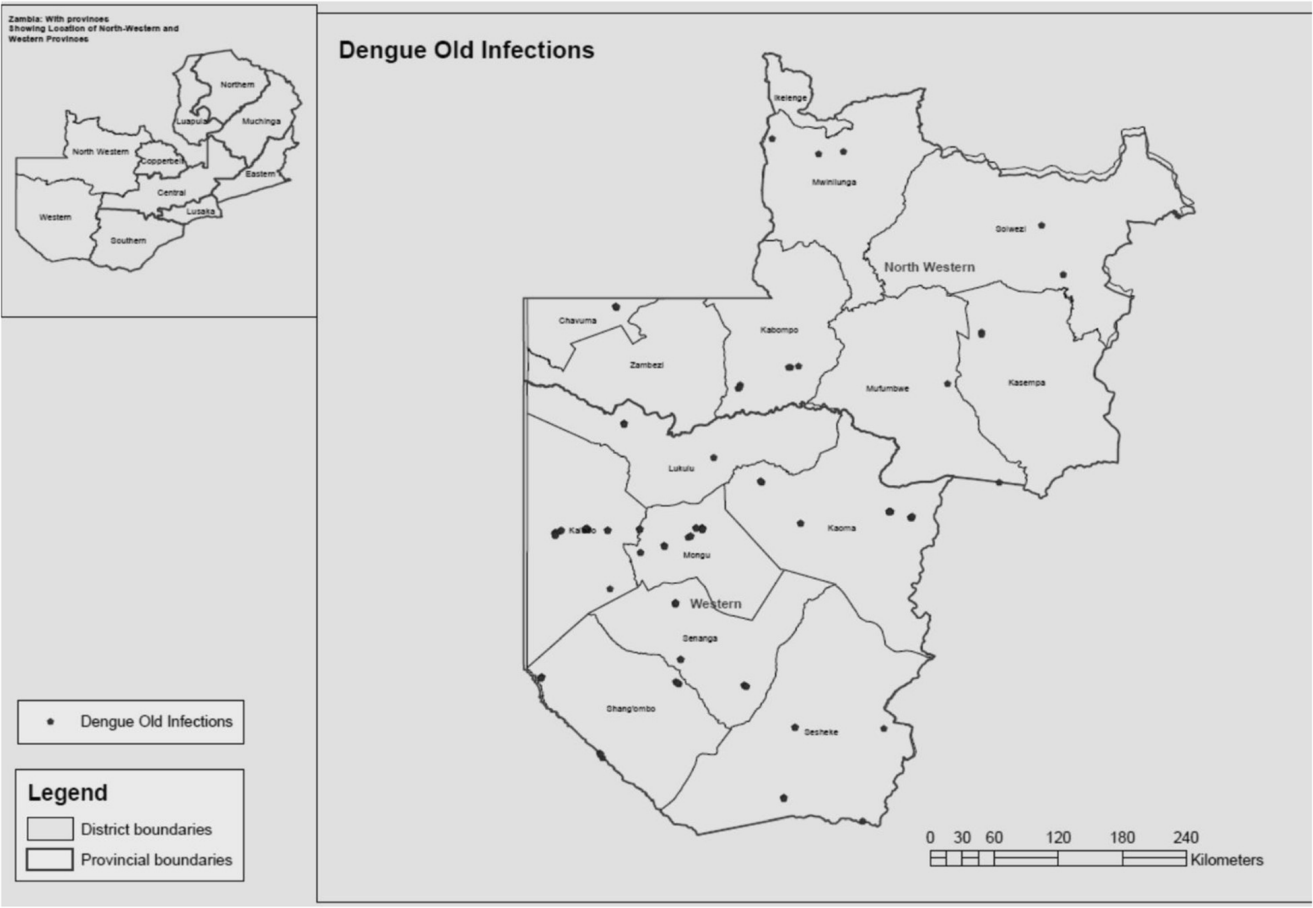
Spatial distribution of Dengue specific IgG positive.

A total of 3,624 persons were sampled for dengue virus infection of whom 23.9% were in the 5–14 years age group. Of the total persons sampled 53.3% were female. Most of the persons in the survey attained at least primary education (47.6%). Male participants tended to be more educated than female participants (p < 0.001). No significant difference was observed between sex and dengue virus infection (p = 1.000). Overall, 4.1% of the participants were found to have had previous dengue infection. These results are shown in Table 1. The special distribution of previous dengue infection was widely spread in the study area (Figure 1).

**Table 1:** Sample description for North-Western and Western provinces for dengue virus infection

**Factor**	**Total**	**Male**	**Female**
	**n (%)**	**n (%)**	**n (%)**
Age (years)	[*x*^2^ = 10.41, p = 0.065]		
<5	335 (9.4)	155 (9.3)	178 (9.4)
5-14	853 (23.9)	424 (25.4)	429 (22.6)
15-24	738 (20.6)	344 (20.6)	392 (20.6)
25-34	593 (16.6)	245 (14.7)	348 (18.3)
35-44	460 (12.9)	214 (12.8)	245 (12.9)
45+	596 (16.7)	286 (17.1)	310 (16.3)
Sex			
Male	1669 (46.7)	-	-
Female	1902 (53.3)	-	-
Education	[*x*^2^ = 37.47, p < 0.001]		
None	748 (21.5)	312 (19.0)	444 (23.6)
Primary	1680 (47.6)	741 (45.1)	937 (49.8)
Secondary or higher	1091 (30.9)	590 (35.9)	500 (26.6)
Roof type	[*x*^2^ = 5.30, p = 0.071]		
Grass	2180 (61.0)	1007 (60.3)	1171 (61.7)
Iron sheet	1302 (35.9)	629 (37.7)	671 (35.3)
Asbestos	91 (2.5)	33 (2.0)	57 (3.0)
Dengue virus Infection	[*x*^2^ = 0.01, p = 1.000]		
Yes	149 (4.1)	69 (4.1)	78 (4.1)
No	3475 (95.9)	1599 (95.9)	1824 (95.9)
Occupation	[*x*^2^ = 245.60, p < 0.001]		
House wife/husband	307 (8.7)	18 (1.1)	289 (15.4)
Farming	1233 (35.1)	573 (35.1)	659 (35.1)
Other	387 (11.0)	232 (14.2)	155 (8.3)
Student	1590 (45.2)	811 (49.6)	775 (41.3)

The bivariate analysis (Table 2) reveals that there was an association between age, education, use of mosquito net, roof type and visit to Angola on one hand and dengue virus infection on the other. In multivariate analysis (Table 2), age, roof type, use of ITNs and visitation to Angola remained significantly associated with dengue virus infection. The association of age with dengue infection shows that participants below 5 years of age were 63% (AOR = 0.37; 95% CI [0.16, 0.86]) less likely to be infected with dengue compared to those aged 45 years or older. A significant association was also observed between grass thatched roofing and dengue infection (AOR = 2.28; 95% CI [1.15,4.53]). The respondents who used ITNs were 21% (AOR = 1.21; 95% CI [1.01,1.44]) more likely to be infected with dengue infection compared to those who did not use ITNs. Persons who visited Angola were 73% (AOR = 1.73; 95% CI [1.27, 2.35]) more likely to be infected with dengue virus compared to those who did not visit Angola.

**Table 2:** Factors associated with Dengue virus infection in logistic regression analysis for North-Western and Western provinces

**Factor**	**OR (95% CI)**	**AOR (95% CI)**
Age (years)		
<5	0.41 (0.20, 0.88)	0.37 (0.16, 0.86)
5-14	0.79 (0.53, 1.17)	0.92 (0.62, 1.38)
15-24	0.92 (0.62, 1.36)	0.98 (0.65, 1.47)
25-34	1.35 (0.93, 1.97)	1.38 (0.94, 2.03)
35-44	0.98 (0.62, 1.56)	0.94 (0.59, 1.50)
45+	1	1
Sex		
Male	1.01 (0.85, 1.19)	-
Female	1	
Education		
None	0.85 (0.63, 1.14)	-
Primary	1.29 (1.03, 1.61)	-
Secondary or higher	1	
Use of an Insecticide Treated Net		
Yes	1.20 (1.01, 1.43)	1.21 (1.01, 1.44)
No	1	1
Insecticide Residual Spraying		
Yes	0,99 (0.80, 1.21)	-
No	1	
Visited Congo DRC		
Yes	0.49 (0.18, 1.30)	-
No	1	
Visited Angola		
Yes	2.00 (1.49, 2.68)	1.73 (1.27, 2.35)
No	1	1
Roof type		
Grass	2.33 (1.18, 4.60)	2.28 (1.15, 4.53)
Iron sheet	0.94 (0.46, 1.90)	0.98 (0.49, 1.99)
Asbestos	1	1
Occupation		
House wife/husband	1.01 (0.65, 1.57)	-
Farming	1.37 (1.05, 1.79)	
Other	1.18 (0.80, 1.73)	
Student	1	

## Discussion

This is the first study that provides evidence on circulation of dengue infection and its correlates in Zambia. Furthermore there is no reported case of clinical dengue infection in Zambia. The results in this study indicate a sero-prevalence of dengue specific IgG antibodies of 4.1%.

Multivariate analyses revealed age, roof type, use of ITNs and visit to Angola as factors associated with dengue infection. The population under five years of age were less likely to have dengue infection, while those who travelled to Angola were more likely to have dengue infection. The relationship between age and risk of dengue has not been consistent. According to Egger and Coleman, the risk for age in relation to classic dengue fever had never been quantified. Using data from clinical patients, Egger and Coleman showed that the risk of classical dengue after primary dengue increased with age [26]. Other studies have revealed that classic dengue is primarily common among older children and adults [6,24]. In a letter to the editor, Cavalcanti et al. indicated that epidemiologic characteristics of Dengue differ by age [27]. The age related association in our study is consistent with other studies that show dengue infection being more common among older children and adults [27].

The association of roof type with dengue infection are inconsistent in studies of seroprevalence of Dengue fever and other flaviviruses. A study on rural Cameroonian populations indicates the odds of dengue seropositivity were significantly lower among individuals with grass or thatched roofs versus those individuals with corrugated tin roofs with unfinished ceilings [28]. However our findings are consistent with a Kenyan study that indicate corrugated metal and thatch roofing were associated with increased odds of antibodies against seropositivity to DEN-2 [29]. We may assume that roofing type may be an important risk factor in exposure to infected mosquitoes.

Our findings in the study reveal that up to 21% of the respondents using ITNs were more likely to be infected with Dengue fever. This is contrast to the findings in Haiti which suggest that ITNs may have protected their study subjects against dengue transmission [30]. We may assume that factors that could have contributed to the risk of Dengue infection in the Zambian population under study include the activities of the vector, *Aedes aegypti*, that of the human host and a possible bias in answering the questionnaire on use of ITNs. *Aedes aegypti* commonly bites during the day [31] and therefore the use of ITNs would not be expected to provide a barrier between the humans and this Dengue fever transmitting vector. Considering the outdoor activities participated in during the day including farming, fishing, and socialising, the population may be at risk of being bitten by the vector.

Although there is evidence of dengue circulating and being endemic in Africa [12-14,18] including Zambia’s neighbours, evidence of dengue virus in Zambia has neither been confirmed nor documented except for a confirmed case of a European traveller/expatriate who was in Zambia between 1987 and 1993 [15]. The disease in Africa, including Zambia is probably not or under reported for various reasons including low awareness of disease among health providers, lack of relevant diagnostic systems and poor surveillance [15].

There is evidence of ongoing dengue outbreaks in Africa in Angola, Democratic Republic of Congo, Mozambique, Tanzania and Seychelles. Two of these countries, Democratic Republic of Congo and Tanzania are neighbouring countries to Zambia. A major drive seen to be contributing to the spread of dengue virus to urban centres around the world is modern transportation and travel to endemic areas [32-35]. The international boundaries within Africa tend to divide villages, with inhabitants of the same family living on either side of the border. This and other factors including small scale trade, and search for medical facilities necessitates frequent travels across the border.

Considering the proximity of North-Western and Western provinces to Angola and a significant association of dengue infection with travel to Angola, we may assume that the outbreak in Angola may have affected the Zambian population in the two study sites.

This study may be limited by the fact that plaque reduction neutralization tests (PRNTs) to increase the specificity of ELISA testing and reduce potential cross reactivity were not performed. However, the IgG capture-ELISA test used is a suitable tool to detecting dengue IgG antibodies in large epidemiologic surveys [36] although caution must be taken knowing that cross-reactivity may occur with other Flavivirus antibodies [37].

## Conclusion

This study provides the first evidence of dengue virus circulation in North-Western and Western provinces of Zambia. It is important that surveillance activities for dengue and diagnostic systems are expanded and strengthened, nationwide in order to capture information related to dengue virus and other flaviviruses. This may reveal the fact that dengue should be considered as a disease of public health importance.

## Competing interests

All authors have approved the submission and declare that they have no competing interests.

## Authors’ contributions

MLM-L – participated in conception, design, data collection, analysis, interpretation of data and drafting of the manuscript. SS – participated in the conception, design, collection of data, analysis, interpretation of data, drafted and critical revision of manuscript. MM – participated in data collection, analysis of data, interpretation of data and critical revision of the manuscript. IM-N - participated in conception, design, data collection, analysis, interpretation of data and critical review of the manuscript. FM - participated in conception, design, data collection, analysis, interpretation of data and drafting of the manuscript. PS – was involved in the conception, design, data collection, analysis, interpretation of data and drafting the manuscript. CM – was involved in the conception and design of the study and critically revised the manuscript. EC – was involved in the conception, design, interpretation of data and critical revision of manuscript. PM – participated in the conception, design, interpretation of data, and critical revision of the manuscript. OAB – was involved in the study during its conception, design, analysis, interpretation of data, drafting the article and revising it critically. All authors read and approved the final manuscript.
